# Assessment of utilisation of government programmes and services by pregnant women in India

**DOI:** 10.1371/journal.pone.0285715

**Published:** 2023-10-05

**Authors:** Balwant Singh Mehta, Ruby Alambusha, Archa Misra, Nidhi Mehta, Aditi Madan

**Affiliations:** 1 Institute for Human Development, Delhi, India; 2 Institute of Economic Growth, North Campus, Delhi University, Delhi, India; 3 Market Xcel Data Matrix Pvt. Ltd., New Delhi, India; 4 The Vision, Lucknow, India; SRM Institute of Science and Technology, INDIA

## Abstract

**Background:**

Since the implementation of various maternal health programs, Maternal Mortality Ratio (MMR) has significantly declined in India through improvements in maternal health services. However, inequality persists at the regional and socio-economic levels. In light of this, the present study aims to assess the existing regional disparities in utilising various government initiatives for safe motherhood in India.

**Methods:**

National-level datasets such as National Family and Health Surveys (NFHS-3 (2005–06); NFHS-4 (2015–16) and NFHS-5(2019–21); Health Management Information System (HMIS), 2019–20; Sample Registrar System (SRS), 2001–2018) were used in the study. In addition, composite Index and inequality measures (Range, Ratio, and Gini) were calculated to examine inequality. At the same time, the Pearson correlation was used to investigate the correlation between various components of maternal health services and Maternal Mortality Rate (MMR).

**Results:**

The composite index score (0.65) reflects that India is still far behind the targets of the utilisation of maternal health care services. Within the utilisation of services, the Gini coefficient reveals that the least inequality was recorded in skilled birth assistance deliveries (0.03) and institutional deliveries (0.04). In contrast, the highest inequality was recorded in receiving Iron and Folic Acid (IFA) Tablets for 100 days (0.19) and four Antenatal Care (ANC) visits (0.13) among selected states. Based on the composite score for maternal health utilisation, Kerala, Tamil Nadu, Andhra Pradesh, Odisha, and Delhi were amongst the best performers, whereas Bihar, Jharkhand, Uttar Pradesh, and Assam were amongst the worst performers.

**Conclusion:**

This indicates that the government’s single-minded focus on enhancing institutional deliveries and skilled health-assisted deliveries has detracted from other essential interventions related to maternal health. Therefore, the states with the utilisation of maternal services need to initiate immediate action to increase the ANC and Post-natal Care (PNC utilisation with more attention towards better implementation of existing ANC programmes by the government.

## Background

Maternal health defines women’s health during pregnancy, childbirth, and postpartum [1]. Improvements in maternal health indicate women’s empowerment and socio-economic development in society. However, an estimated 800 women die daily due to pregnancy and childbirth complications. Safe motherhood continues to be at the forefront of national and global health policies, where the reduction of maternal mortality is placed as one of the key monitoring indicators in Millennium Development Goals (MDGs) and Sustainable Development Goals (SDGs). Despite this, the global progress of maternal mortality has been slow and uneven [2, 3], where a majority of maternal deaths were noted to occur in developing and under-developed regions of Sub-Saharan Africa (68 per cent) and South Asia (19 per cent). Many governments, non-governmental organisations and international organisations have implemented conditional cash transfer programs, also known as Demand-Side Financing (DSF), to increase facility-based deliveries [4–9] in developing countries.

Many studies consistently found that these programmes have successfully increased Maternal Care Utilization and Maternal Mortality Reduction (MMR) [10–15]. According to UNICEF, maternal deaths declined from 451 thousand in 2000 to 295 thousand in 2017. MMR also declined from 342 per 100,000 live births in 2000 to 211 per 100 000 live birth in 2017, with a 2.9 per cent annual average rate of reduction. Across the regions, South Asia has recorded the most remarkable fall in MMR, i.e. from 395 per 100,000 live births to 163 per 100,000 live births during the last two decades (Table 1). However, the MMR remains high in most sub-Saharan African and South Asian countries [2].

**Table 1:** Maternal Mortality Ratio (MMR) (number of maternal deaths per 1000,000 live birth) [2].

Year	Global	South Asia	India
2000	451	395	370
2017	295	163	145

India is the largest country in South Asia and the second most populous country in the world with a sizeable reproductive cohort, where one in every four global maternal death occurs (113 thousand out of 451 thousand). It has experienced a significant fall in MMR (370 in 2000 to 145 in 2017) [2], as shown in Table 1. For over three decades, many SDG targets have been set to reduce maternal deaths in India, with SDG-3 mainly targets reducing MMR to 70 per 100,000 live births by 2030 [3].

As most maternal deaths are avoidable with timely interventions during pregnancy and childbirth. The Government of India (GoI) introduced several programmes to ensure that all pregnant women receive essential and professional antenatal care services for safe motherhood. Under these programmes, the government actively promoted institutional delivery, Ante Natal Care (ANC) and Post Natal Care (PNC), to curb the high MMR. However, there has been considerable improvement in the MMR, maternal health remains a challenge in India. This could be attributed to several factors, an important non-utilisation or under-utilisation of safe-motherhood services. Given the massive investment by the Indian government and the high uptake of maternal services in recent years, it is critical to utilisation and non-utilisation of safe-motherhood services in the country. Previous studies highlight the negligence of complete antenatal care, deficiency in skilled birth attendants during delivery, and high cost of maternal healthcare resulting in high MMR [16–25].

Against this backdrop, the Indian government policies and interventions on maternal healthcare services, the programme’s implementation, utilisation, and underutilisation need a regular assessment to understand its effects on the performance indicators of maternal health. However, in recent years, there has been a shortage of studies analysing utilisation of government safe maternal health programs in detail. This is very relevant considering the significance of maternal health in the context of SDGs and the fact that many countries worldwide are working towards meeting the SDG targets. India’s experience with the recent safe motherhood programs, including cash transfer and non-cash services, can be emulated by other countries with a similar stage of development. Thus, the present paper examines the status and coverage of maternal healthcare services and utilisation across 22 major states after implementing several new initiatives in recent years.

This paper is divided into four sections. Section one discusses the background and objective, the second section presents the material and methods, the third section focuses on results and discussions, and the last section provides conclusion and key policy suggestions.

## Material and methods

### Data sources and indicators

Various secondary sources have been used, including the National Health Management Information System (HMIS), 2019–20; Sample Registrar System (SRS), Registrar General, Census of India, 2001–2018; National Family and Health Surveys (NFHS-3 (2005–06); NFHS-4 (2015–16) and NFHS-5 (2019–21). In addition, relevant information was collected from various published research studies, journal articles and other websites [26–30] as shown in Table 2.

**Table 2:** Data source and variables.

Data Sources	Variables
National Health Management Information System (HMIS) Annual Reports [30, 41]	Total expected pregnant women, Pregnant women registered for ANC
Sample Registrar System (SRS) bulletins for the years, 2001–03, 2004–06, 2007–09, 2010–12, 2014–16, 2015–17 and 2016–18, Registrar General, Census of India [29, 42]	Maternal Mortality Ratio
National Family and Health Surveys (NFHS): NFHS-3, (2005–06); NFHS-4(3015–16) and NFHS-5(2019–21), Fact Sheets [26–28, 43–45]	1^st^ trimester ANC, 4 ANC; Tetanus toxoid vaccine (TT2+); Post-natal; Iron Folic Acid (IFA) 100 days, Iron Folic Acid (IFA) 180 days, Anaemia; Intuitional birth, public facility, skilled health personnel, skilled health personnel (home); mother received post-natal care; children received post-natal care -2 days of delivery

Utilisation of maternal health services has been measured through seven indicators, i.e. 1^st^ trimester ANC, ANC 4; TT2+; Post-natal care-2 days; Intuitional delivery; skilled Assisted delivery; FBA tablets 100+ days, while the maternal mortality ratio has been used as an outcome indicator.

### Statistical analysis

Statistical techniques were used to analyse the regional variation and association between the utilisation of anti-natal services and outcome indicators.

### Composite index

The composite score for maternal health per utilisation is an unweighted average normalised value of seven indicators namely, 1^st^ trimester ANC, ANC 4; TT2+; Post-natal care-2 days; Intuitional delivery; skilled Assisted delivery; FBA tablets 100+ days. All the values are in percentage and uni-directional, which means the higher values indicate better performance. To arrive at the composite index, the indicators have normalised using a procedure applied by the United Nations Development Programme (UNDP) for the Human Development Index (HDI), namely, the max-min method: Y_i_ = (X_i_ -X_min_) / (X_max_- X_min_), where Y_i_ normalised indicator for state i, X_i_ is the corresponded-normalisation value of the selected indicator, and X_max_ and X_min_ are the maximum and minimum values of the same indicator. The composite index value is the average of normalised values of the indicators, which varies between 0 and 1, with 0 being the worst and 1 being the best-performing state in the utilisation of maternal health services. The data for indicators were collected using NHFS-3 (2005–06), NFHS-4 (2015–16), and NFHS-5 (2019–21) factsheets [26–28, 43–45].

### Inequity measures

The intra-state disparities in maternal health services were examined by computing range and range ratio. The ranges were calculated by taking the difference between the highest and lowest value in a set of numbers, while the range ratios were computed by dividing the maximum value by the minimum values. Further, the inequality in distribution was quantified by Gini Coefficient. The Gini coefficient of 0 expresses perfect equality, where all values are the same, and 1 depicts perfect inequality. The classical definition of Gini appears in the notation of the theory of relative mean difference as given below:

$\sum_{i=1}^{n}\sum_{j=1}^{n}|xi-xj|/{2n}^{2}\bar{x}$, where x is an observed value, n is the number of values observed, and x bar is the mean value.

### Correlation

The association between the variable is tested by using Pearson correlation, which measures the strength and direction of the linear relationship between the variables. A p-value less than 0.05 is considered statistically significant. The person correlation coefficient values range from -1 to +1, -1 indicating a perfect negative correlation, +1 showing a perfect positive correlation, and 0 indicating no correlation.

### Ethics statement

This study is based on secondary data which is available in the public domain for research use.

### Statistical software

In this paper, all the statistical analyses were performed using the statistical software STATA version 20.0. The Gini, range, and range ratio statistics were computed using Stata commands.

## Results and discussion

### Maternity care programmes: Coverage

In post-independent India, maternal health has been a part of the family welfare programmes with a special focus on family planning services, aiming to slow population growth, protect mothers’ health by delaying pregnancy and facilitate birth spacing. In the 1990s, there was a paradigm policy shift concerning the high MMR and women’s reproductive rights. As a result, the Child Survival and Safe Motherhood (CSSM) programme was introduced in 1992, followed by National Maternity Benefit Scheme (NMBS) under the National Social Assistance Scheme in 1995 to provide monetary benefits for pregnancy up to the first two live births, and Reproductive and Child Health-I (RCH-I) programme in 1997 emphasised the provision of Antenatal Care (ANC), safe home deliveries and institutional deliveries. However, the improvement in institutional births remained slow. Therefore, the government introduced, Reproductive and Child Health-II (RCH-II), emphasising coverage and quality of antenatal and postnatal care and engaged the private sector in the provision of maternity care [31–33].

The RCH-II programme was launched along with National Rural Health Mission (NRHM). This Indian health system reform promised to increase the budget allocations to rural India in the national budget and to make architectural changes to the health system. One of the key components of NRHM was initiating a conditional cash transfer scheme called- Janani Suraksha Yojana (JSY) by modifying the NMBS—to provide financial support to women undergoing institutional delivery. The JSY was launched in 2005 as a nationwide flagship programme, under the umbrella of the NRHM, for providing monetary incentives to women upon delivery in public or accredited private health facilities. It was launched due to the low uptake of facility-based care for childbirth during the previous safe motherhood programmes (i.e., CSSM and RCH-I).

The underlying assumption of the JSY is that if women deliver in health facilities instead of at home, they will be able to access skilled and emergency obstetric care in the event of complications. The JSY programme marked a significant change in the Indian government’s strategies to make childbirths safe compared to previous programmes on safe motherhood. While previous efforts focused mainly on strengthening the supply side through, for example, the training of birth attendants and improvement of health facilities’ functionality, the JSY programme looked at addressing the demand-side barriers preventing women from accessing health facilities for childbirth [31–34].

Notwithstanding this, five years later, India continued to show marked deficits in healthcare service use. Previous evaluation of JSY provides some insights into why it largely failed to redress the country’s dismal maternal and child health record. While it substantially increased deliveries in public health institutions, it did not reduce maternal and new-born mortality. Some of the programme features considered responsible for JSY’s limited success include, firstly, its narrow focus on institutional delivery; secondly, short time interval coverage; thirdly, cash incentives for all live births; and lastly, a lack of qualitatively adequate healthcare infrastructure. To complement JSY, an additional programme, Indira Gandhi Matritva Sahyog Yojana (IGMSY), was introduced in 2011 to cover a time interval of nine months around delivery, including additional supply-side financing of health personnel, which was later renamed Pradhan Mantri Matru Vandana Yojana (PMMVY) in 2017.

In contrast to JSY, IGMSY covers all childbirths by a woman, and cash transfer was paid for only the first two live births of women aged 19 and older. However, after renaming the scheme in 2017, the eligibility criteria under PMMVY were further restricted to the first live birth only. In addition, the GoI introduced several programs to ensure quality healthcare and nutrition services for pregnant women [19, 21, 35–37] as shown in Table 3 below:

**Table 3:** Maternity benefits in India: A snapshot.

Scheme/Launched year	Entitlement	Eligibility	Assistance	Impact and Beneficiary in a million) (2019-20/2020-21)
Janani Suraksha Yojana (JSY), 2005 [46]	INR 6,000/-	Poor/SC/ST pregnant women delivering in public health centres or accredited private institutions are eligible.	Cash transfer (conditional)	10.7 million/10.0 million
Janani Shishu Suraksha Karyakaram (JSSK), 2011 [47].	Free transport from home to institution, between facilities in case of a referral and drop back home.	All pregnant women delivering in public health facilities.	Free of cost, including caesarean section. (conditional)	0.49 million/0.54 million million
Mission Indradhanush, 2014 [48].	Immunisation	All pregnant women.	Free of cost (Universal)	9.2** million
Pradhan Mantri Surakshit Matritva Abhiyan (PMSMA), 2016 [49].	Comprehensive antenatal services in the 2^nd^ and 3^rd^-trimester women on the 9^th^ day of every month.	All pregnant women.	Free (no cost) (Universal)	30.2 million pregnant women received ANC, and over 2.6 million were identified as high-risk pregnancies and referred to a specialist or a higher health facility for appropriate care.
Pradhan Mantri Matru Vandana Yojana (PMMVY), 2017 [50].	INR 5,000/- in three instalments during pregnancy.	All pregnant women, first live children only delivered in public health centres.	Cash transfer (conditional)	7.1 million/6.4 million beneficiaries. This scheme is criticized for excluding women with more than one child.
POSAN Abhiyan, 2017 [51].	Rations 600 cal, 18/20 gms of protein, during pregnancy and until six months after childbirth.	All pregnant and lactating women.	In-kind transfers (conditional)	16.9 million/16.7 million beneficiaries.

** Total coverage since inception.

### Maternity care programmes: Utilisation

The utilization of maternal health services has been analysed under three broad headings: ANC services, Delivery services and PNC services. In contrast, the outcome variable, namely MMR, has been analysed under two sections: all India and regional differences to assess the impact of maternal health care services utilisation.

### Ante Natal Care (ANC)

**Fig 1:**
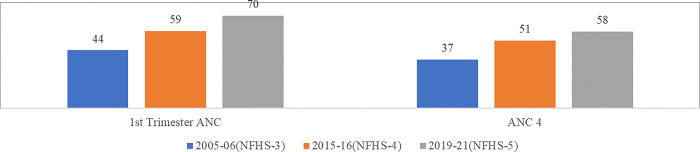
Ante-natal health care (in %) [26–28, 43–45].

Antenatal Care refers to the healthcare services received by a woman during pregnancy. They are advised to follow a proper diet, undergo regular antenatal check-ups, and receive counselling for family planning. Women are also provided with TT immunization, IFA, Calcium and Albendazole tablets, and proper treatment for any complication [1, 20]. According to HMIS, at the national level, the total number of expected pregnant women for 2019–20 was estimated at 29.93 million [30]. Around 97 per cent of the estimated pregnant women registered for ANC. Among the total registered pregnant women, about 72 per cent registered within the first trimester of their pregnancy. When a pregnant woman registers herself with a health facility within the first 12 weeks of her pregnancy for antenatal care, it is considered first-trimester registration. All pregnant women are recommended to go for their first antenatal check-up in the first Trimester to identify and manage any medical complications and screen themselves for any risk factors that may affect the progress and outcome of their pregnancy [1, 21]. The NFHS data also reveals that almost 70 per cent of pregnant women had an antenatal check-up in the first Trimester of 2019–21 during their last pregnancy in the five years preceding the survey. The proportion of women who had reported antenatal check-ups in the first Trimester increased by 15 percentage points from 44 to 59 per cent during 2006–2016 and improved by 11 percentage points to 70 per cent in 2019–21 (Fig 1).

It is argued that many women in the reproductive age group (15–49 years) from lower socio-economic groups are compelled to work for survival. In such conditions, attendance at the antenatal clinic may result in a loss of daily earnings. Consequently, it is difficult for them to attend the antenatal clinic so often [15–18, 20]. In such cases, a minimum of 4 visits covering the pregnancy should be the target. The World Health Organisation (WHO) also recommends a minimum of four antenatal visits based on a review of the effectiveness of different models of antenatal care [1, 20]. More than half (58 per cent) of pregnant women made at least four antenatal care visits in 2019–21, which increased by 15 percentage points from 37 to 51 per cent during 2006–2016 and further improved by seven percentage points to 58 per cent in 2019–21 (Fig 1). Antenatal care visit during the 1^st^ Trimester was higher than four or more ANC visits, which increased more than the ANC visits during the recent period from 2016–2021. This improvement in ANC uptake among Indian women has been attributed to several maternity care schemes launched recently such as Pradhan Mantri Matru Vandana Yojana (PMMVY), Pradhan Mantri Surakshit Matritva Abhiyan (PMSMA), and POSAN Abhiyan.

### Anaemia: Iron Folic Acid (IFA) and tetanus injection

**Fig 2:**
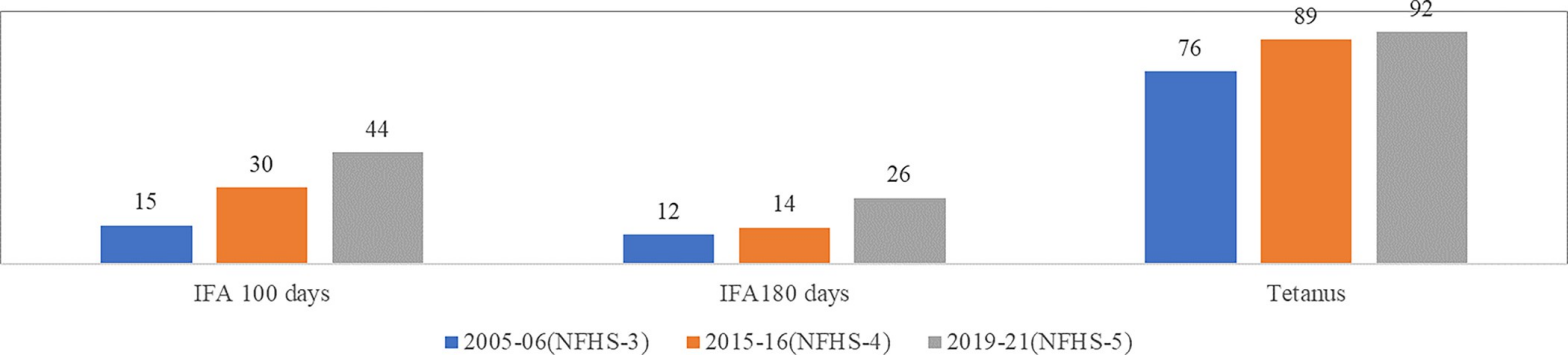
IFA tablets and tetanus injection (in %) [26–28, 43–45].

Oral Iron Folic Acid (IFA) supplementation with 30 mg to 60 mg of elemental iron and 0.4 mg of folic acid was recommended for pregnant women to prevent maternal Anaemia, puerperal sepsis, low birth weight, and preterm birth. As per the norm, every pregnant woman should avail of 100 or more IFA tablets and two or more TT injections [1, 21]. However, only around one-fourth (26%) of pregnant women consumed IFA for 180 days or more, which increased by only two percentage points from 12 per cent in 2005–06 to 14 per cent in 2015–16 and further to 26 per cent in 2019–21. On the other hand, 44 per cent of pregnant women consumed IFA for 100 days or more when pregnant, which increased from 30 per cent in 2015–16, and 15 per cent in 2005–06 (Fig 2).

All the women giving birth and their new-born babies should be protected against tetanus. If the mother is not immunised with the correct number of doses of tetanus toxoid vaccine (TT2+), it puts her and her new-born infant at risk at the time of delivery [1, 21]. A majority (92 per cent) of pregnant women were protected against neonatal tetanus in 2019–21, which increased from 76 per cent in 2005–06, and 89 per cent in 2015–16. However, as per IHMS data, the TT2+ prevalence among total ANC registration is significantly less, 71 per cent of pregnant women had been protected by one tetanus, and 62 per cent by two tetanus in 2019–21 [33].

The lower rate of IFA consumption and TT2+ injection is reflected in the higher prevalence of Anaemia, especially iron-deficiency Anaemia among pregnant women. More than half (52 per cent) of pregnant women were anaemic in 2019–21, which declined by six percentage points from 58 per cent in 2005–06 but increased by two percentage points in recent years (50 per cent in 2015–16).

### Institutional delivery

**Fig 3:**
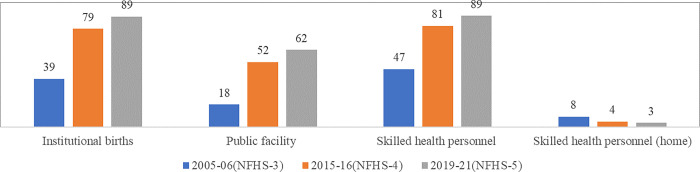
Institutional and skilled health care delivery (in %) [26–28, 43–45].

The role of institutional delivery in improving maternal health in India is undeniable and widely documented. The proportion of institutional deliveries was recorded at 90 per cent in 2019–21, which went up almost two and a half times from 39 in 2005–06, and 10 percentage points in 2015–16 (Fig 3). Birth attended by skilled personnel simultaneously almost doubled from 47 per cent in 2004–05 to 89 per cent in 2019–21, and delivery in the public facility increased by more than three times from 18 per cent to 62 per cent during the same period. On the other hand, the government’s efforts to institutionalise births are reflected through a reduction of birth attended by skilled health personnel at home from 8 per cent in 2005–06 to 3 per cent in 2019–21. Further, the studies show that households incur huge out-of-pocket expenditures on institutional delivery services in India [17, 34]. In this context, the recent years’ results are encouraging, where the average out-of-pocket spending per delivery in a public health facility has reduced from INR 3197 in 2015–16 to INR 2916 in 2019–21 at nominal prices. This may be an outcome of the increasing penetration of government-safe motherhood schemes such as JSY.

### Post-natal care

**Fig 4:**
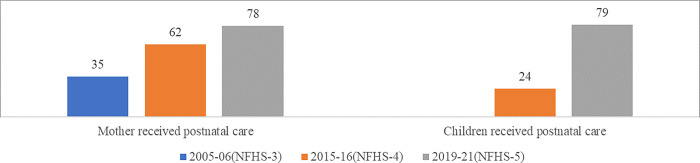
Post-natal care—2 days of delivery (in %) [26–28, 43–45].

Post-natal check-up after delivery is as essential as ANC services. It gives required medical attention to women and new-borns and reduces most complications resulting in maternal and neonatal mortality [1]. As per the guidelines of NHM, all women were asked whether they had received PNC check-ups soon after their delivery, specifically on the 3^rd^ and 7^th^ day of delivery [38, 39]. As a result, over three-fourths (78%) of the mothers received post-natal care from skilled health personnel (doctor/nurse/LHV/ANM/midwife/other health) within two days of delivery in 2019–21, which improved more than twice from 37 per cent in 2005–06 and increased by 16 percentage points from 62 per cent in 2015–16. Similarly, 79 per cent of new-born children received post-natal care from skilled health personnel within two days of delivery in 2019–21, which increased more than three times from 24 per cent in 2015–16 (Fig 4). These data underscore the better utilisation of national safe motherhood programmes in recent years.

The results reveal that the increase in the coverage of ANC services has not been as much as that in institutional delivery services. Around 4 out of 10 pregnant women in the country were not even getting the basic recommended 4 ANC, while 4 out of 10 pregnant women did not consume IFA tablets for 100 days or more. There was also a rural-urban difference in maternal health facilities in ANC services, consumption of IFA tablets for 100+ days, institutional deliveries, and post-natal care (Table 4).

**Table 4:** Maternal health service by rural-urban (in %) [28].

	1st Trimester ANC	ANC 4	TT2+	IFA Tablets 100+ Days	Institutional Delivery	Skilled Assistance	PNC-2 Days
All	70	58	92	44	89	89	78
Urban	68	54	93	54	94	94	85
Rural	76	68	92	40	87	88	75

### Regional disparities: Utilisation of health services

Given the size of the country, national estimates conceal wide rural-urban and regional variations. Nevertheless, overall utilisation of maternal healthcare services improved in most of the major states (22 states) during the past years. However, the magnitude of improvement varied across the states and in different service components. Therefore, a composite index was constructed using seven key indicators for each state using UNDP methods [40] as discussed in the methodology section, to examine the differences across the major states in terms of performance. A similar composite index called the achievements of childcare services index had been used in the past to compare the regional performance in childcare services [32]. These states are further ranked based on the composite index to examine the relative performance of overall maternal health services over the years (2016–2021) (S3 Table). The states are divided into three major groups based on the composite index: (i) top five best-performing states (0.75+), (ii) moderate-performing states (0.50 to 0.74), and (iii) worst-performing states (less than 0.50). At both time points (20015–16 and 2019–21), Kerala, Tamil Nadu, Andhra Pradesh, Odisha, and Delhi are among the best performers. On the other hand, Bihar, Jharkhand, Uttar Pradesh, and Assam are amongst the worst performers. The rest of the states, Telangana, Gujarat, Haryana, Himachal Pradesh, Jammu and Kashmir, West Bengal, Karnataka, Maharashtra, Punjab, Madhya Pradesh, Rajasthan, Chhattisgarh, and Uttarakhand are amongst the moderate performers (Table 5).

**Table 5:** Maternal health service index score in the major states: 2019–21. (Authors generated the estimate employing the NFHS Data [28]).

Performance	States (Composite Index Score Range)
Best (0.75 and above)	Kerala (0.97), Tamil Nadu (0.87), Andhra Pradesh (0.80), Odisha (0.77), Delhi (0.75)
Moderate	Telangana (0.74), Gujarat (0.71), Haryana (0.71), Himachal Pradesh (0.62), Jammu and Kashmir (0.70), West Bengal (0.68), Karnataka(0.71), Maharashtra(0.65), Punjab(0.64), Madhya Pradesh(0.66), Rajasthan(0.66), Chhattisgarh(0.55), Uttarakhand (0.52)
Worst (less than 0.50)	Assam (0.47), Uttar Pradesh (0.37), Jharkhand (0.31), Bihar (0.13)

**Fig 5:**
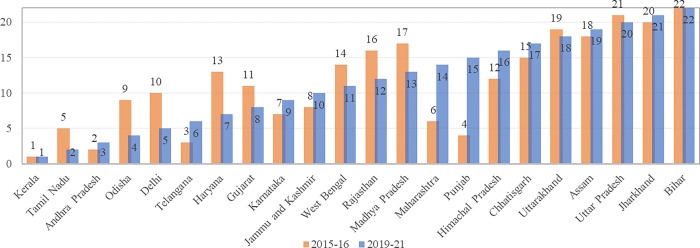
Index value of maternal health care: Major states: 2016–21. (Authors generated the estimate employing the NFHS Data [27, 28]).

Based on the composite index ranking, among 22 major states in India, ten states recorded deterioration in their relative performance(ranks) during 2016–2021: Haryana (13–7), Delhi (10–5), Odisha (9–4), Rajasthan (16–12), Madhya Pradesh (17–13), Tamil Nadu (5–2), Andhra Pradesh(3–2), West Bengal(14–11), Gujarat(11–8), Uttarakhand(19–18), Uttar Pradesh(21–20) recorded an improvement in their performance. Contrarily ten states: Punjab (4–15), Maharashtra (6–14), Himachal Pradesh, (12–16), Chhattisgarh (14–17), Telangana (3–6), Jammu & Kashmir (8–10), Karnataka (7–9), Chhattisgarh, Assam (19–18) and Jharkhand (21–20) (Fig 5).

There are two states, one at the top (Kerala) and another at the bottom (Bihar), which have shown no improvement in their respective ranking. In particular, the worst-performing states, i.e., states having the lowest composite index value (Assam, Uttar Pradesh, Jharkhand, and Bihar), are still far behind the levels of best-performing states, i.e., states having the highest composite index value (Kerala and Tamil Nadu). This analysis reveals that the regional patterns in maternal health services have remained largely unchanged over the last 4–5 years, with few states performing much better than the others (S1A and S1B Table).

Utilisation of different components of ANC—antenatal health check-ups, at least four ANC visits, at least one ANC visit in the first Trimester, and IFA consumption for at least 100+ days, and receiving TT2+, was recorded lowest in Bihar, Jharkhand, and Uttar Pradesh in 2019–21. On the other hand, most of the major Indian states in the country have recorded very high incidences of institutional deliveries and skilled birth assistance deliveries. For example, around 90 per cent of women in 16 out of 22 major states gave birth in health facilities in 2019–21. Also, a similar proportion of women received skilled birth-assisted deliveries in 18 states of India (S1A and S1B Table).

Given such wide variations in maternal health services, examining the disparities across states is critical to comprehend whether they are narrowing. For this purpose, three indicators were used: range difference computed as the difference between the highest and lowest value of each health service component; range ratio calculated as a ratio of the highest and lowest value of each service component, and the Gini coefficient. The inequality analysis shows that the range difference was recorded as highest in ANC4 (65) and IFA 100+ days (65) components and lowest in TT2+ component (6) across the major states of India (Table 6). Over the years, the gap reduced to maximum in first trimester ANC (20 points) and TT2+ components (20 points), and minimum in skilled birth assistance deliveries (9 points), and IFA 100 days (-8 points). The range ratio was recorded highest in IFA 100+ days (4.6) and ANC4 (3.6) components, while lowest in institutional delivery (1.3) and skilled birth assistance deliveries (1.3) components. During 2016–2021, ANC4 (2.9 points) and IFA 100 days (2.3 points) services noted the highest reduction in range ratio, while TT2+ (0.3 points) and skilled birth assistance deliveries (0.1 points) showed the slightest drop in range ratio during 2016–2021. The Gini index reveals the least inequality across the states in skilled birth assistance deliveries (0.03) and institutional delivery (0.04). In contrast, the highest Gini index was recorded in IFA 100+ days (0.19), and ANC 4 services (0.13) across the states. The Gini inequality index has declined maximum for ANC 4(0.07 point), and IFA 100+ days (0.06 point) during 2016–2021. In agreement with the previous studies, the present study also showed regional disparities across states that women residing in Southern states of India received better maternal health care services than others. Women in Southern India have higher level of autonomy and better health infrastructure; thereby, they are better able to obtain available maternal care services than the other regions [17, 33].

**Table 6:** Inequality indices: Magnitude of inter-state disparities in maternal health service components: 2016–2021. (Authors generated the estimate employing the NFHS Data [27, 28]).

Inequality	1st Tri ANC	ANC 4	TT2+	IFA 100 Days	Inst. Delivery	Skilled Birth Asst.	PNC-2 Days
*Range difference(H-L)*							
2019–21	41	65	6	65	24	21	36
2015–16	61	76	26	57	38	30	46
*Range ratio(H/L)*							
2019–21	1.8	3.6	1.1	4.6	1.3	1.3	1.6
2015–16	2.8	6.3	1.4	6.9	1.6	1.4	2.1
*Gini*							
2019–21	0.07	0.13	0.01	0.19	0.04	0.03	0.06
2015–16	0.11	0.20	0.03	0.25	0.08	0.06	0.11

### Maternal mortality

The above analysis reveals a substantial improvement in the utilisation of maternal healthcare services during the last one and half decade’s period. It is assumed that higher utilisation of maternal healthcare services may also reflect a lower maternal mortality ratio (MMR). As per WHO, “Maternal death is the death of a woman while pregnant or within 42 days of termination of pregnancy, irrespective of the duration and site of the pregnancy, from any cause related to or aggravated by the pregnancy or its management but not from accidental or incidental causes” [1, 29]. MMR has been defined as the number of maternal deaths during a given time per 100,000 live births during the same period. Target 3.1 of the Sustainable Development Goals (SDG) set by the United Nations aims at reducing the global maternal mortality ratio to less than 70 per 100,000 live births [3].

**Fig 6:**
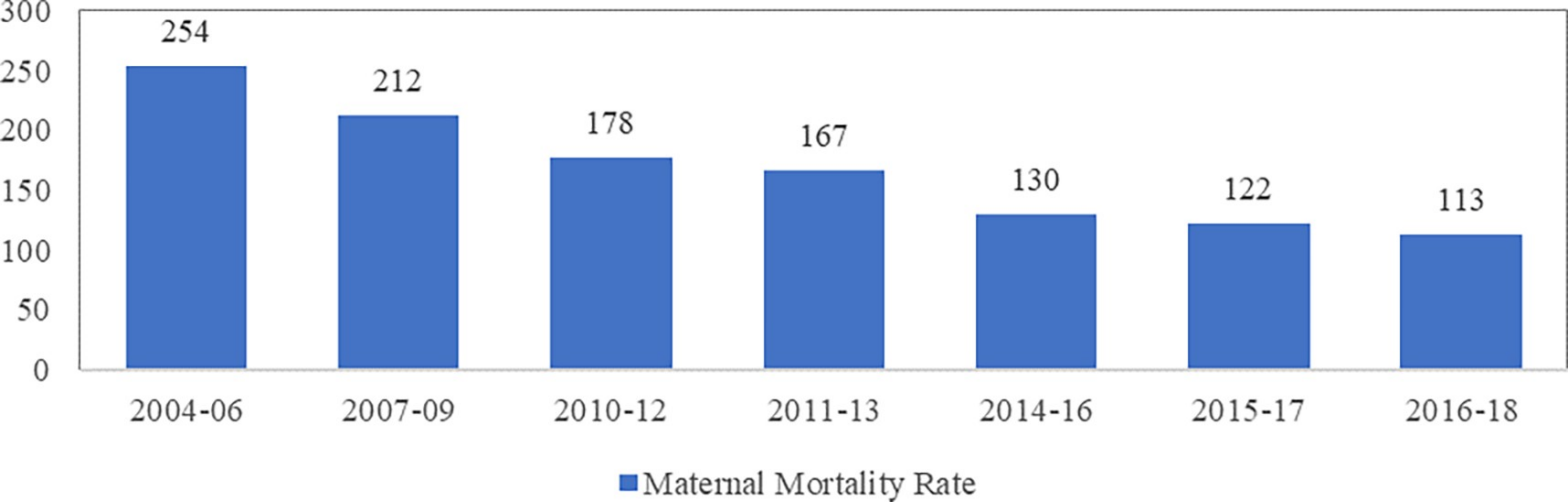
Maternal Mortality Ratio (per 100,000 live births) [29, 42].

The trend analysis presented here is based mainly on the SRS estimates presented in four successive MMR bulletins for 2001–03, 2004–06, 2007–09, 2010–12, 2014–16, 2015–17 and 2016–18. It is visible from Fig 6 that the MMR in India continues to be very high (113 maternal deaths per 100,000 live births), and that the reduction in MMR has slowed down in recent times. MMR has declined from 178 per 100,000 live births in 2010–12, to 254 per 100,000 live births in 2004–06. It is argued that several factors have helped the country to achieve a lower level of MMR, such as (i) the government’s concerted push to increase access to quality maternal health services (ii) women entering into marriage at an older age, and being more literate than ever, enabling a to better control over their reproductive choices and decision making in their interests and, (iii) government’s substantive efforts to facilitate positive engagement of public and private health care providers, in particular, Pradhan Mantri Surakshit Matritva Abhiyan (PMSMA) with significant impact by allowing women’s easy access to ANC. Despite this, India’ MMR still lags behind in meeting the SDG targets [3, 30], which is only higher than Nepal, Bhutan and Afghanistan, and at par with Bangladesh and Pakistan. But still lower than other Asian countries like Indonesia, Thailand, Sri Lanka and China [15, 32]

### Maternal mortality: Regional disparities

**Fig 7:**
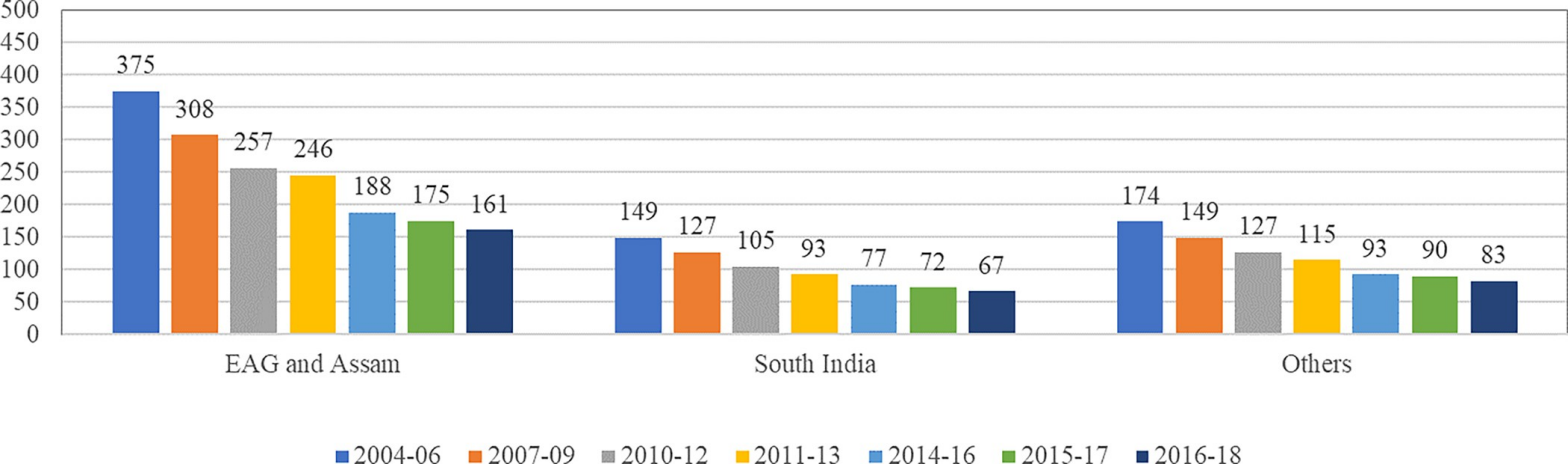
Maternal Mortality Ratio (per 100,000 live births) [29, 42].

The state-level MMR estimates reveal a wide inter-state disparity in the maternal health situation. In addition, there are critical equity concerns, as studies have noted significant inter and intra-state disparities [33]. Across regions, ‘South Indian states’ (67 per 100,000 live births), namely Andhra Pradesh, Telangana, Karnataka, Kerala, and Tamil Nadu, have already achieved the SDG target of MMR below 70 per 100,000 live births by 2030. On the other hand, ‘other states’ (83 per 100,000 live births), namely Gujarat, Haryana, Maharashtra, Punjab, West Bengal, and others, are near to achieving the SDG target. India’s Ministry of Health and Family Welfare has identified ‘Empowered Action Group (EAG) states’ based on high fertility and mortality indicators to facilitate focused efforts to promote the reproductive and child health programmes, which include Bihar, Jharkhand, Madhya Pradesh, Chhattisgarh, Uttar Pradesh, Uttarakhand, Odisha and Rajasthan. These states account for about 48 per cent of India’s population [29, 35]. During the last one and half decade period, MMR has reduced maximum in EAG states and Assam region (214), followed by Others (91), and Southern states (82). However, MMR estimates for EAG and Assam region (161) are still very high and 2.4 times higher than that for Southern states. There is high variation at regional level estimates, with the highest MMR being in EAG states and Assam and the lowest in the Southern States (Fig 7).

This regional-level analysis underlines considerable geographical heterogeneity in MMR across Indian states. To understand the difference across the states, MMR is categorised into three groups, less than 70 per 100,000 live births, 70–139 per 100,000 live births, and greater than or equal to 140 deaths per 100,000 live births. The first cut-off was taken at 70 per 100,000 live births, which is a primary target under SDG-3 for MMR, while the second cut-off is at 140 per 100,000 live births, which is a second target under SDGs. Such categorisation allows for classifying Indian states as those achieved, near to committing, or far from the achievable SDG target 3.1. The estimates indicate in Table 7 shows that five out of the major 20 states have MMR less than 70 per 100,000 live births, namely Kerala (43), Maharashtra (46), Tamil Nadu (60), Telangana (63), and Andhra Pradesh (65) indicating already achieved the SDG target of 2030. Seven states have MMR in the range of 70–139 per 100,000 live births, namely Jharkhand (71), Gujarat (75), Haryana (91), Karnataka (92), West Bengal (98), Uttarakhand (99), and Punjab (129). Among the rest 20 states, the highest MMR above 140 per 100,000 live births is found in 6 states, namely Bihar (149), Odisha (150), Rajasthan (164), Madhya Pradesh (173), Uttar Pradesh (197), and Assam (215). These states also recorded high lifetime risk and confidence interval upper level (S2 Table).

**Table 7:** Performance of Maternal Mortality Rate: States (2016–18). (Authors generated the estimate employing the SRS Data [29, 42]).

Performance	States (MMR)
High (less than 70)	Kerala (43), Maharashtra (46), Tamil Nadu (60), Telangana (63), Andhra Pradesh (65)
Moderate (70–139)	Jharkhand (71), Gujarat (75), Haryana (91), Karnataka (92), West Bengal (98), Uttarakhand (99), Punjab (129)
Poor (above 140)	Bihar (149), Odisha (150), Rajasthan (164), Madhya Pradesh (173), Uttar Pradesh (197), Assam (215)

Table 8 shows the inequality in MMR across the states. The range shows significant (172) regional differences in MMR, which was highest in Assam (215 per 100,000 live births) and lowest in Kerala (43 per 100,000 live births) (Table 7). Although the gaps between the extremes have reduced significantly from 385 per 100,000 live births in 2004–06 to 172 per 100,000 live births in 2016–18, the range ratio has stabilised and hovered around five during the same period. In other words, the MMR of the worst-performing states has remained five times greater than the best-performing states during the study period. Nevertheless, the magnitude of inter-state disparities has reduced over the years, but it remains high, which is also confirmed by the Gini coefficient for the same years.

**Table 8:** Inequality measures: Magnitude of inter-state disparities in MMR. (Authors generated the estimate employing the SRS Data [29, 42]).

	2004–06	2007–09	2010–12	2011–13	2014–16	2015–17	2016–18
Range	385	309	262	239	191	187	172
Range ratio	5.05	4.81	4.97	4.92	5.15	5.45	5.00
Gini	0.27	0.25	0.25	0.26	0.25	0.19	0.20

### Bivariate analysis: Utilisation and outcome

The association between MMR and maternal health care services components is presented in Table 9 through correlation coefficients (R-value) and respective P value. The health service components are statistically significant and negatively correlated with MMR. For example, there is a statistically significant negative correlation of first Trimester ANC (R = -0.586, P = 0.008), ANC4 (R = -0.546, P = 0.017), IFA100 days (R = -0.531, P = 0.019), PNC-2 days (R = -0.487, P = 0.034), and Institutional delivery (R = -0.487, P = 0.035) with MMR. On the other hand, the correlation between TT2+ and skilled health assistance deliveries with MMR is negative but not statistically significant. Hence, the correlation analysis indicates that first trimester ANC, ANC4, IFA 100+ days, PNC-2 days, and institutional delivery are associated with the reduction of MMR.

**Table 9:** Components of maternal health services correlation with MMR (P value < 0.05): 2019–21. (Authors generated the estimate employing the NFHS and SRS Data([28, 29, 42]).

Correlates of MMR	R-value	P value
1st Trimester ANC	-.586**	.008
ANC4	-.541*	.017
TT2+	.336	.159
IFA100+ days	-.531*	.019
Institutional delivery	-.487*	.035
Skilled birth-assisted deliveries	-.506	.027
PNC-2 days	-.489*	.034

**. Correlation is significant at the 0.01 level (2-tailed).

*. Correlation is significant at the 0.05 level (2-tailed).

However, the correlation results show no statistically significant association between birth attended by skilled health personnel and MMR. Looking at this finding in conjunction with previous studies, which showed an unexpected relationship with both infant mortality and maternal mortality, it suggests that it may be due to a considerable number of women rushing to skilled health assistance deliveries when complications arise; most often a majority of them remain without receiving complete and quality antenatal care [33]. Thus, risky deliveries contribute to more deaths at home, even from skilled health personnel, compared to institutional deliveries.

Based on the above analysis, this paper highlights following critical issues regarding the utilisation of government maternal health care services in India.

The institutional delivery and antenatal care visits have increased substantially, but still, every four out of ten pregnant women did not make a recommended minimum of four ANC visits. There is a very low consumption rate of IFA among pregnant women, as only four, out of ten pregnant women consumed IFA for 180 days or more. On the other hand, six out of ten pregnant women were protected by two tetanus. This resulted in a high prevalence of Anaemia, where around half of the pregnant women were anaemic. There are wide regional differences in the utilisation of maternal healthcare services in India. In particular, the EAG states such as Bihar, Jharkhand, Uttar Pradesh, and Assam performed worst among the major states. This also confirms the earlier findings, which revealed that women from the poorest households, less educated, and residing in EAG states and Assam have significantly higher MMR than non-EAG states [16]. Furthermore, the regional disparity is relatively higher in the case of ANC 4 and IFA consumption, while less in institutional delivery, and skilled birth assistance. This points to whether the government’s single-minded focus on enhancing institutional deliveries and skilled health assistance deliveries has taken the attention away from other essential interventions for maternal health. Women receiving post-natal care (PNC), and consumption of IFA is much less than the incidence of institutional delivery.

The mothers receiving post-natal care within the first two days after delivery is critical for mothers. But many maternal deaths also occur in the first six weeks after delivery, yet this remains the most neglected phase in providing quality maternal care. There is no significant negative association between birth attended by skilled health personnel and MMR. This demonstrates that women delivering with assistance from skilled health personnel are not automatically receiving sufficient care. Although the government has initiated several new health schemes, India’s government health care financing remains inadequate. It is one of the lowest at 1.28 per cent of GDP in the regions, and even less than Nepal, Sri Lanka, Thailand, and Indonesia [15, 16].

## Conclusion

India has made decent progress in maternal health service utilisation in the last one and half decades. This progress could be attributed to Janani Suraksha Yojana (JSY) and several new initiatives such as Pradhan Mantri Surakshit Matritva Abhiyaan (PMSMA), Surakshit Matritva Aashwasan (SUMAN), POSHAN Abhiyaan, and LaQshya Programme among others. However, the achievement of universal utilisation of maternal services is still a long journey.

From a policy perspective, the finding suggests that continuous awareness campaigns and counselling for young married couples and students at educational and health institutions may help increase the antenatal care visits and consumption of IFA among pregnant women. The women especially targeting those residing in poor households and rural areas required special attention and affordable services. In addition, it is argued that maternity entitlement for women under JSY is not sufficient, the average spending on maternity care (US$ 258) is more than ten times than the JSY entitlement [19]. So, there is a need to increase the JSY entitlement to address the high out-of-pocket expenditure that many women incur for maternity care.

The length of stay following childbirth indicates the quality of post-natal care. Therefore, it is crucial to maintain an adequate length of stay at a facility after the birth. Thus, the government should strengthen the JSY scheme to improve delivery care and provide effective post-natal care by promoting sufficient length of stay at facilities.

At the regional or state levels with low utilisation of maternal services, the EAG states need to initiate immediate action to increase the ANC and PNC-2 utilisation and consumption of IFA. In addition, they have to focus more on better implementation of the existing government maternal healthcare programmes through continuous monitoring and evaluation. This is also necessary to meet India’s SDG-3 target and eliminate preventable maternal mortality.

The potential beneficiaries of our study would be public health practitioners, policy-makers, and non-government organisations working in maternal and child health care. In addition, future research and further studies can be conducted at the district level using unit-level data from NFHS and HMIS to identify the reasons behind the performance of states at the micro-level, which can guide the state governments and other stakeholders for better planning and implementation at the district level.

## Supporting information

S1 Table A: Maternal Health Care (in per cent (col. 1–7)) Indicators, Index Score and Rank by Major States: 2019–21 [29]. B: Maternal Health Care (in per cent (col. 1–7) Indicators, Index Score and Rank by Major States: 2015–16 [28].
(DOCX)

S2 Table Trend in Maternal Mortality Rates (MMR), CI, Lifetime Risk, and Change in MMR:2004–2018 [30]. (DOCX)

S3 Table (DOCX)

## References

[1] World Health Organization. United Nations Population Fund, World Bank, United Nations. Population Division & United Nations Children’s Fund (‎UNICEF)‎. (‎2014)‎. Trends in maternal mortality: 1990 to 2013: estimates by WHO, UNICEF, UNFPA, The World Bank and the United Nations Population Division: executive summary. World Health Organization; 2014. https://apps.who.int/iris/handle/10665/112697

[2] UNICEF, Monitoring the situation of children and women, maternal mortality, September 2021. https://data.unicef.org/dv_index/?q=MMR.

[3] United Nations. Sustainable Development Goal-3 (SDGs-3): Ensure healthy lives and promote well-being for all at all ages, 2015. http://www.un.org/sustainabledevelopment/health/.

[4] Bhutta ZA, Das JK, Bahl R, Lawn JE, Salam RA, Paul VK, et al. Can available interventions end preventable deaths in mothers, new-born babies, and stillbirths, and at what cost? The Lancet. 2014 Jul 26;384(9940):347–70. 10.1016/S0140-6736(14)60792-3 24853604

[5] Souza J P, Gülmezoglu A M, Vogel J, Carroli G, Lumbiganon P. Moving beyond essential interventions for reduction of maternal: a cross-sectional study. The Lancet 381 (9879): 1747–55. doi: 10.1016/S0140-6736(13)60686-8 23683641

[6] Kuwawenaruwa A, Mtei G, Baraka J, Tani K. Implementing demand side targeting mechanisms for maternal and child health-experiences from national health insurance fund program in Rungwe District, Tanzania. Globalization and Health. 2016 Dec;12(1):1–2. doi: 10.1186/s12992-016-0180-x 27480025 PMC4970262

[7] Rahman MM, Pallikadavath S. How much do conditional cash transfers increase the utilization of maternal and child health care services? New evidence from Janani Suraksha Yojana in India. Economics & Human Biology. 2018 Sep 1; 31:164–83. doi: 10.1016/j.ehb.2018.08.007 30265897

[8] Skiles MP, Curtis SL, Basinga P, Angeles G, Thirumurthy H. The effect of performance-based financing on illness, care-seeking and treatment among children: an impact evaluation in Rwanda. BMC Health Services Research. 2015 Jun;15(1):1–1. doi: 10.1186/s12913-015-1033-7 26369410 PMC4570690

[9] Yang L, Sun L, Wen L, Zhang H, Li C, Hanson K, et al. Financing strategies to improve essential public health equalization and its effects in China. International Journal for Equity in Health. 2016 Dec;15(1):1–2. doi: 10.1186/s12939-016-0482-x 27905941 PMC5134004

[10] Barber SL, Gertler PJ. Empowering women to obtain high-quality care: evidence from an evaluation of Mexico’s conditional cash transfer programme. Health Policy and Planning. 2009 Jan 1;24(1):18–25. doi: 10.1093/heapol/czn039 19022854 PMC2724849

[11] De Brauw A, Hoddinott J. Must conditional cash transfer programs be conditioned to be effective? The impact of conditioning transfers on school enrollment in Mexico. Journal of Development Economics. 2011 Nov 1;96(2):359–70. 10.1016/j.jdeveco.2010.08.014

[12] Kiplagat S, Coudray MS, Ravi K, Jayakrishna P, Krupp K, Arun A, et al. Evaluating a Conditional Cash Transfer Scheme in a Maternal Health Care Utilization Program Among Rural Pregnant Women in Mysore District, India. Women’s Health Reports. 2020 Jun 1;1(1):159–66. doi: 10.1089/whr.2019.0021 32617535 PMC7325491

[13] Ng M, Misra A, Diwan V, Agnani M, Levin-Rector A, De Costa A. An assessment of the impact of the JSY cash transfer program on maternal mortality reduction in Madhya Pradesh, India. Global Health Action. 2014 Dec 1;7(1):24939. doi: 10.3402/gha.v7.24939 25476929 PMC4256523

[14] Powell-Jackson T, Hanson K. Financial incentives for maternal health: impact of a national programme in Nepal. Journal of Health Economics. 2012 Jan 1;31(1):271–84. doi: 10.1016/j.jhealeco.2011.10.010 22112695

[15] Von Haaren P, Klonner S. Lessons learned? Intended and unintended effects of India’s second‐generation maternal cash transfer scheme. Health Economics. 2021 Sep;30(10):2468–86. doi: 10.1002/hec.4390 34278651

[16] Bhatia M, Dwivedi LK, Banerjee K, Bansal A, Ranjan M, Dixit P. Pro-poor policies and improvements in maternal health outcomes in India. BMC pregnancy and childbirth. 2021 Dec;21(1):1–3. 10.1186/s12884-021-03839-w 34011316 PMC8135986

[17] Deepak C, Jauhari N, Dhungana H. A study on utilization of maternal health services and factors influencing the utilization in urban slums of Lucknow. International Journal of Medicine and Public Health. 2018;8(2). doi: 10.5530/ijmedph.2018.2.17

[18] Gabrysch S, Campbell OM. Still too far to walk: literature review of the determinants of delivery service use. BMC Pregnancy and Childbirth. 2009 Dec;9(1):1–8. doi: 10.1186/1471-2393-9-34 19671156 PMC2744662

[19] Goli S, Rammohan A, Pradhan J. High spending on maternity care in India: what are the factors explaining it?. PloS one. 2016 Jun 24;11(6):e0156437. doi: 10.1371/journal.pone.0156437 27341520 PMC4920397

[20] Kumar G, Choudhary TS, Srivastava A, Upadhyay RP, Taneja S, Bahl R, et al. Utilisation, equity and determinants of full antenatal care in India: analysis from the National Family Health Survey 4. BMC Pregnancy and Childbirth. 2019 Dec;19(1):1–9 doi: 10.1186/s12884-019-2473-6 31488080 PMC6727513

[21] Paul Sohini. Inequality in the utilisation of maternal healthcare services: Evidence from Indian states. Economic & Political Weekly. Nov 2020. 55(45). 37–44.

[22] Perkins M, Brazier E, Themmen E, Bassane B, Diallo D, Mutunga A, et al. Out-of-pocket costs for facility-based maternity care in three African countries. Health Policy and Planning. 2009 Jul 1;24(4):289–300. doi: 10.1093/heapol/czp013 19346273 PMC2699243

[23] Rao KD, Kachwaha S, Kaplan A, Bishai D. Not just money: what mothers value in conditional cash transfer programs in India. BMJ Global Health. 2020 Oct 1;5(10):e003033. doi: 10.1136/bmjgh-2020-003033 33087391 PMC7580051

[24] Thind A, Mohani A, Banerjee K, Hagigi F. Where to deliver? Analysis of choice of delivery location from a national survey in India. BMC Public Health. 2008 Dec;8(1):1–8. doi: 10.1186/1471-2458-8-29 18218093 PMC2248571

[25] Vora KS, Mavalankar DV, Ramani KV, Upadhyaya M, Sharma B, Iyengar S, et al. Maternal health situation in India: a case study. Journal of Health, Population, and Nutrition. 2009 Apr;27(2):184. doi: 10.3329/jhpn.v27i2.3363 19489415 PMC2761784

[26] International Institute for Population Sciences (IIPS). National Family Health Survey (NFHS-3) Report, 2005–06. http://rchiips.org/nfhs/factsheet.shtml.

[27] International Institute for Population Sciences (IIPS). National Family Health Survey (NFHS-4) Report, 2015–16. http://rchiips.org/nfhs/factsheet_NFHS-4.shtml

[28] International Institute for Population Sciences (IIPS). National Family Health Survey (NFHS-5) Report, 2019–21. http://rchiips.org/nfhs/factsheet_NFHS-5.shtml

[29] Bulletin on Maternal Mortality in India. New Delhi: Sample Registration System, Office of Registrar General, Vital Statistics Division, India. https://censusindia.gov.in/census.website/data/SRSSTAT

[30] HIMS, Annual Report, 2019–20. an analytical report, statistics division ministry of health & family welfare government of India. Available: https://hmis.nhp.gov.in/downloadfile?filepath=publications/Other/HMIS%20Annual%202019-20%20Report.pdf

[31] Barber SL, Gertler PJ. Empowering women to obtain high quality care: evidence from an evaluation of Mexico’s conditional cash transfer programme. Health Policy and Planning. 2009 Jan 1;24(1):18–25. doi: 10.1093/heapol/czn039 19022854 PMC2724849

[32] Dreze J, Khera R. Regional patterns of human and child deprivation in India. Economic and Political Weekly. 2012 Sep 29: 47(39). 42–49.

[33] Banerjee Soumik, John Priya, and Singh Sanjeev. Stairway to death: maternal mortality beyond numbers. Economic and Political Weekly. 2013 August 3. 48 (31): 123–30.

[34] Montgomery AL, Ram U, Kumar R, Jha P, Million Death Study Collaborators. Maternal mortality in India: causes and healthcare service use based on a nationally representative survey. PloS one. 2014 Jan 15;9(1):e83331. doi: 10.1371/journal.pone.0083331 24454701 PMC3893075

[35] Kumar C, Singh PK, Rai RK. Under-five mortality in high focus states in India: a district level geospatial analysis. Plos one. 2012 May 18;7(5):e37515. doi: 10.1371/journal.pone.0037515 22629412 PMC3356406

[36] Nandi A, Laxminarayan R. The unintended effects of cash transfers on fertility: Evidence from the Safe Motherhood Scheme in India. Journal of Population Economics. 2016 Apr;29(2):457–91. doi: 10.1007/S00148-015-0576-6

[37] Lahariya C. A brief history of vaccines & vaccination in India. The Indian Journal of Medical Research. 2014 Apr;139(4):491. 24927336 PMC4078488

[38] Singh A, Padmadas SS, Mishra US, Pallikadavath S, Johnson FA, et al. (2012) Socio-economic inequalities in the Use of Post-natal Care in India. PLoS ONE 7(5): e37037. doi: 10.1371/journal.pone.0037037 10.1371/journal.pone.0037037 22623976 PMC3356397

[39] Chaturvedi S, Upadhyay S, De Costa A. Competence of birth attendants at providing emergency obstetric care under India’s JSY conditional cash transfer program for institutional delivery: an assessment using case vignettes in Madhya Pradesh province. BMC pregnancy and childbirth. 2014 Dec;14(1):1–1. doi: 10.1186/1471-2393-14-174 24885817 PMC4075933

[40] United Nations Development Programme (UNDP). Technical Notes: Calculating the Human Development Indices—Graphical Presentation. Available: https://hdr.undp.org/system/files/documents//technical-notes-calculating-human-development-indices.pdf

[41] HMIS-Health Management Information System. https://hmis.nhp.gov.in/#!/

[42] Government of India | Office of the Registrar General & Census Commissioner, India. https://censusindia.gov.in/census.website/data/SRSSTAT

[43] National Family Health Survey 3. http://rchiips.org/nfhs/factsheet.shtml

[44] National Family Health Survey 4. http://rchiips.org/nfhs/factsheet_NFHS-4.shtml

[45] National Family Health Survey 5. National Family Health Survey (NFHS-5) (rchiips.org)

[46] Janani Suraksha Yojana (JSY) | National Health Portal Of India. https://www.nhp.gov.in/janani-suraksha-yojana-jsy-_pg

[47] Janani Shishu Suraksha Karyakaram (JSSK): National Health Mission. https://nhm.gov.in/index4.php?lang=1&level=0&linkid=150&lid=171

[48] Mission Indradhanush | National Health Portal Of India. https://www.nhp.gov.in/mission-indradhanush1_pg

[49] Pradhan Mantri Surakshit Matritva Abhiyan. In: PMSMA. https://pmsma.nhp.gov.in/10.4103/jfmpc.jfmpc_1636_21 PMC9254766 35800511

[50] Pradhan Mantri Matru Vandana Yojna | Ministry of Women & Child Development. https://wcd.nic.in/schemes/pradhan-mantri-matru-vandana-yojana

[51] POSHAN Abhiyaan. http://poshanabhiyaan.gov.in/#/

