# Supplementary material for: Assessment of utilisation of government programmes and services by pregnant women in India
Source: PLoS One. 2023 Oct 5;18(10):e0285715. doi: 10.1371/journal.pone.0285715 (PMC10553210; doi:10.1371/journal.pone.0285715)
Supplement: S1 Table — A: Maternal Health Care (in per cent (col. 1–7)) Indicators, Index Score and Rank by Major States: 2019–21 [29]. B: Maternal Health Care (in per cent (col. 1–7) Indicators, Index Score and Rank by Major States: 2015–16 [28]. (DOCX) [file pone.0285715.s001.docx]

S1A: Maternal Health Care (in per cent (col. 1-7)) Indicators, Index Score and Rank by Major States: 2019-21[29]

| **State** | **1st Tri**  **ANC** | **ANC 4** | **TT2+** | **IFA**  **100 Days** | **Inst.**  **Delivery** | **Skill Birth**  **Asst.** | **PNC-2**  **Days** | **Index**  **Score** | **Rank** |
| --- | --- | --- | --- | --- | --- | --- | --- | --- | --- |
|  | **(1)** | **(2)** | **(3)** | **(4)** | **(5)** | **(6)** | **(7)** |  |  |
| Andhra Pradesh | 81.7 | 68 | 93 | 70 | 97 | 96 | 91 | 0.80 | 3 |
| Assam | 64 | 51 | 95 | 48 | 84 | 86 | 65 | 0.47 | 19 |
| Bihar | 53 | 25 | 90 | 18 | 76 | 79 | 57 | 0.13 | 22 |
| Chhattisgarh | 66 | 60 | 92 | 45 | 86 | 89 | 84 | 0.55 | 17 |
| Delhi | 76 | 77 | 93 | 69 | 92 | 93 | 85 | 0.75 | 5 |
| Gujarat | 79 | 77 | 89 | 60 | 94 | 93 | 90 | 0.71 | 8 |
| Haryana | 85 | 60 | 91 | 51 | 95 | 94 | 91 | 0.71 | 7 |
| Himachal Pradesh | 72 | 70 | 90 | 67 | 88 | 87 | 86 | 0.62 | 16 |
| Jammu and Kashmir | 87 | 81 | 92 | 30 | 92 | 95 | 84 | 0.70 | 10 |
| Jharkhand | 68 | 39 | 91 | 28 | 76 | 83 | 69 | 0.31 | 21 |
| Karnataka | 71 | 71 | 94 | 45 | 97 | 94 | 87 | 0.71 | 9 |
| Kerala | 94 | 79 | 95 | 80 | 100 | 100 | 93 | 0.97 | 1 |
| Madhya Pradesh | 75 | 58 | 95 | 51 | 91 | 89 | 84 | 0.66 | 13 |
| Maharashtra | 71 | 70 | 90 | 48 | 95 | 94 | 85 | 0.65 | 14 |
| Odisha | 77 | 78 | 95 | 61 | 92 | 92 | 88 | 0.77 | 4 |
| Punjab | 69 | 59 | 90 | 55 | 94 | 96 | 86 | 0.64 | 15 |
| Rajasthan | 76 | 55 | 93 | 34 | 95 | 96 | 85 | 0.66 | 12 |
| Tamil Nadu | 77 | 90 | 90 | 83 | 100 | 100 | 93 | 0.87 | 2 |
| Telangana | 89 | 70 | 90 | 58 | 97 | 94 | 88 | 0.74 | 6 |
| Uttar Pradesh | 63 | 42 | 92 | 22 | 83 | 85 | 72 | 0.37 | 20 |
| Uttarakhand | 69 | 62 | 94 | 47 | 83 | 84 | 78 | 0.52 | 18 |
| West Bengal | 73 | 76 | 95 | 63 | 92 | 94 | 68 | 0.68 | 11 |

S1B: Maternal Health Care (in per cent (col. 1-7) Indicators, Index Score and Rank by Major States: 2015-16[28]

| **State** | **1st Tri**  **ANC** | **ANC 4** | **TT2+** | **IFA**  **100 Days** | **Inst.**  **Delivery** | **Skill Birth**  **Asst.** | **PNC-2**  **Days** | **Index**  **Score** | **Rank** |
| --- | --- | --- | --- | --- | --- | --- | --- | --- | --- |
|  | **(1)** | **(2)** | **(3)** | **(4)** | **(5)** | **(6)** | **(7)** |  |  |
| Andhra Pradesh | 82.4 | 76.3 | 95.0 | 56.2 | 91.6 | 92.2 | 79.7 | 0.82 | 2 |
| Assam | 55.1 | 46.5 | 89.9 | 32.0 | 70.6 | 74.3 | 54.0 | 0.40 | 18 |
| Bihar | 34.6 | 14.4 | 89.6 | 9.7 | 63.8 | 70.0 | 42.3 | 0.16 | 22 |
| Chhattisgarh | 70.8 | 59.1 | 94.3 | 30.3 | 70.2 | 78.0 | 63.6 | 0.51 | 15 |
| Delhi | 63.3 | 68.6 | 89.9 | 49.9 | 84.4 | 86.9 | 62.6 | 0.63 | 10 |
| Gujarat | 73.9 | 70.6 | 86.8 | 36.8 | 88.7 | 87.3 | 63.4 | 0.63 | 11 |
| Haryana | 63.2 | 45.1 | 92.3 | 32.5 | 80.5 | 84.7 | 67.3 | 0.54 | 13 |
| Himachal Pradesh | 70.5 | 69.1 | 86.3 | 49.4 | 76.4 | 78.9 | 70.2 | 0.59 | 12 |
| Jammu and Kashmir | 76.8 | 81.4 | 87.5 | 30.2 | 85.7 | 87.6 | 74.9 | 0.66 | 8 |
| Jharkhand | 52.0 | 30.3 | 91.8 | 15.3 | 61.9 | 69.6 | 44.4 | 0.25 | 20 |
| Karnataka | 66.0 | 70.3 | 88.3 | 45.3 | 94.3 | 93.9 | 65.6 | 0.69 | 7 |
| Kerala | 95.1 | 90.2 | 96.5 | 67.1 | 99.9 | 100.0 | 88.7 | 1.00 | 1 |
| Madhya Pradesh | 53.1 | 35.7 | 89.8 | 23.6 | 80.8 | 78.1 | 55.0 | 0.40 | 17 |
| Maharashtra | 67.6 | 72.2 | 90.4 | 40.6 | 90.3 | 91.1 | 78.5 | 0.71 | 6 |
| Odisha | 64.1 | 62.0 | 94.5 | 36.5 | 85.4 | 86.6 | 73.3 | 0.64 | 9 |
| Punjab | 75.6 | 68.5 | 92.9 | 42.6 | 90.5 | 94.1 | 87.2 | 0.77 | 4 |
| Rajasthan | 63.0 | 38.5 | 89.7 | 17.3 | 84.0 | 86.6 | 63.7 | 0.49 | 16 |
| Tamil Nadu | 64.0 | 81.2 | 71.0 | 64.0 | 99.0 | 99.3 | 74.1 | 0.73 | 5 |
| Telangana | 83.1 | 75.0 | 89.1 | 52.8 | 91.5 | 91.4 | 81.8 | 0.79 | 3 |
| Uttar Pradesh | 45.9 | 26.4 | 86.6 | 12.9 | 67.8 | 70.4 | 54.0 | 0.25 | 21 |
| Uttarakhand | 53.5 | 30.9 | 91.4 | 24.9 | 68.6 | 71.2 | 54.8 | 0.33 | 19 |
| West Bengal | 54.9 | 76.5 | 95.4 | 28.1 | 75.2 | 81.7 | 61.1 | 0.54 | 14 |
