# Supplementary material for: Assessment of utilisation of government programmes and services by pregnant women in India
Source: PLoS One. 2023 Oct 5;18(10):e0285715. doi: 10.1371/journal.pone.0285715 (PMC10553210; doi:10.1371/journal.pone.0285715)
Supplement: S2 Table — (DOCX) [file pone.0285715.s002.docx]

S2: Trend in Maternal Mortality Rates (MMR), CI, Lifetime Risk, and Change in MMR:2004-2018[30]

| **State/Region** | **2004-**  **06** | **2007-**  **09** | **2010-**  **12** | **2011-**  **13** | **2014-**  **16** | **2015-**  **17** | **2016-**  **18** | **95% CI**  **(2016-18)** | **Lifetime**  **Risk^[[1]](#footnote-1)^** | **Change**  **(2006-16)** | **Change**  **(2006-18)** |
| --- | --- | --- | --- | --- | --- | --- | --- | --- | --- | --- | --- |
| India | 254 | 212 | 178 | 167 | 130 | 122 | 113 | (103-123) | 0.3 | 124 | 141 |
| Assam | 480 | 390 | 328 | 300 | 237 | 229 | 215 | (133-297) | 0.5 | 243 | 265 |
| Bihar | 312 | 261 | 219 | 208 | 165 | 165 | 149 | (104-194) | 0.5 | 147 | 163 |
| Jharkhand | - | - | - | - | - | 76 | 71 | (20-123) | 0.2 |  |  |
| Madhya Pradesh | 335 | 269 | 230 | 221 | 173 | 188 | 173 | (126-221) | 0.6 | 162 | 162 |
| Chhattisgarh |  |  |  |  |  | 141 | 159 | (69-249) | 0.4 |  |  |
| Odisha | 303 | 258 | 235 | 222 | 180 | 168 | 150 | (96-205) | 0.3 | 123 | 153 |
| Rajasthan | 388 | 318 | 255 | 244 | 199 | 186 | 164 | (112-215) | 0.5 | 189 | 224 |
| Uttar Pradesh | 440 | 359 | 292 | 285 | 201 | 216 | 197 | (152-241) | 0.6 | 239 | 243 |
| Uttarakhand | - | - | - | - | - | 89 | 99 | (49-150) | 0.2 |  |  |
| EAG and Sub-Total | 375 | 308 | 257 | 246 | 188 | 175 | 161 | (143-180) | 0.5 | 187 | 214 |
| Andhra Pradesh | 154 | 134 | 110 | 92 | 74 | 74 | 65 | (26-104) | 0.1 | 80 | 89 |
| Telangana |  |  |  |  |  | 76 | 63 | (16-110) | 0.1 |  |  |
| Karnataka | 213 | 178 | 144 | 133 | 108 | 97 | 92 | (53-131) | 0.2 | 105 | 121 |
| Kerala | 95 | 81 | 66 | 61 | 46 | 42 | 43 | (10-77) | 0.1 | 49 | 52 |
| Tamil Nadu | 111 | 97 | 90 | 79 | 66 | 63 | 60 | (29-92) | 0.1 | 45 | 51 |
| South Sub-Total | 149 | 127 | 105 | 93 | 77 | 72 | 67 | (50-84) | 0.1 | 72 | 82 |
| Gujarat | 160 | 148 | 122 | 112 | 91 | 87 | 75 | (41-109) | 0.2 | 69 | 85 |
| Haryana | 186 | 153 | 146 | 127 | 101 | 98 | 91 | (43-139) | 0.2 | 85 | 95 |
| Maharashtra | 130 | 104 | 87 | 68 | 61 | 55 | 46 | (19-73) | 0.1 | 69 | 84 |
| Punjab | 192 | 172 | 155 | 141 | 122 | 122 | 129 | (56-202) | 0.2 | 70 | 63 |
| West Bengal | 141 | 145 | 117 | 113 | 101 | 94 | 98 | (59-137) | 0.2 | 40 | 43 |
| Other states | 206 | 160 | 136 | 126 | 96 | 96 | 85 | (62-108) | 0.2 | 110 | 121 |
| Other Sub-Total | 174 | 149 | 127 | 115 | 93 | 90 | 83 | (68-97) | 0.2 | 81 | 91 |

1. The life time risk is defined as the probability that at least one women of reproductive age (15-49) will die due to child birth or puerperium assuming that chance of death is uniformly distributed across the entire reproductive span and has been worked out. [↑](#footnote-ref-1)
