# Supplementary material for: Assessment of utilisation of government programmes and services by pregnant women in India
Source: PLoS One. 2023 Oct 5;18(10):e0285715. doi: 10.1371/journal.pone.0285715 (PMC10553210; doi:10.1371/journal.pone.0285715)
Supplement: S3 Table — (DOCX) [file pone.0285715.s003.docx]

**S3.1: Ante-Natal Health Care (in %)** [27-29]

|  | 2005-06(NFHS-3) | 2015-16(NFHS-4) | 2019-21(NFHS-5) |
| --- | --- | --- | --- |
| 1st Trimester ANC | 44 | 59 | 70 |
| ANC 4 | 37 | 51 | 58 |
| IFA consumption at least for 100 days | 15 | 30 | 44 |
| IFA consumption at least for 180 days | 12 | 14 | 26 |
| Protected against tetanus | 76 | 89 | 92 |

**S 3.2: IFA Tablets and Tetanus Injection (in %)** [27-29]

|  | 2005-06(NFHS-3) | 2015-16(NFHS-4) | 2019-21(NFHS-5) |
| --- | --- | --- | --- |
| IFA 100 days | 15 | 30 | 44 |
| IFA180 days | 12 | 14 | 26 |
| Tetanus | 76 | 89 | 92 |

**S 3.3: Institutional and Skilled Health Care Delivery (in %)**[27-29]

|  | 2005-06(NFHS-3) | 2015-16(NFHS-4) | 2019-21(NFHS-5) |
| --- | --- | --- | --- |
| Institutional births | 39 | 79 | 89 |
| Public facility | 18 | 52 | 62 |
| Skilled health personnel | 47 | 81 | 89 |
| Skilled health personnel (home) | 8 | 4 | 3 |

**S 3.4: Post-Natal Care - 2 Days of Delivery (in %)** [27-29]

|  | 2005-06(NFHS-3) | 2015-16(NFHS-4) | 2019-21(NFHS-5) |
| --- | --- | --- | --- |
| Mother received postnatal care | 35 | 62 | 78 |
| Children received postnatal care | - | 24 | 79 |
| Note: (-) indicate data is not available |  |  |  |

**S 5.1: Index Value of Maternal Health Care: Major States (2015–16)** [28]

| State | Actual Value | | | | | | | Normalised value | | | | | | | Index |
| --- | --- | --- | --- | --- | --- | --- | --- | --- | --- | --- | --- | --- | --- | --- | --- |
|  | First Trimester  ANC | ANC 4 | TT2 | IFA  100 Days | Institutional  Delivery | Skilled Assistance | PPC-2 Days | First  Trimester  ANC | ANC 4 | TT2 | IFA  100 Days | Institutional Delivery | Skilled  Assistance | PPC-2  Days |  |
| Andhra Pradesh | 81.7 | 68 | 93 | 70 | 97 | 96 | 91 | 0.73 | 0.66 | 0.77 | 0.81 | 0.88 | 0.84 | 0.93 | 0.80 |
| Assam | 64 | 51 | 95 | 48 | 84 | 86 | 65 | 0.31 | 0.41 | 0.93 | 0.46 | 0.44 | 0.44 | 0.28 | 0.47 |
| Bihar | 53 | 25 | 90 | 18 | 76 | 79 | 57 | 0.06 | 0.02 | 0.46 | 0.01 | 0.15 | 0.16 | 0.07 | 0.13 |
| Chhattisgarh | 66 | 60 | 92 | 45 | 86 | 89 | 84 | 0.36 | 0.55 | 0.69 | 0.43 | 0.49 | 0.55 | 0.76 | 0.55 |
| Delhi | 76 | 77 | 93 | 69 | 92 | 93 | 85 | 0.60 | 0.81 | 0.83 | 0.80 | 0.71 | 0.74 | 0.80 | 0.75 |
| Gujarat | 79 | 77 | 89 | 60 | 94 | 93 | 90 | 0.67 | 0.80 | 0.42 | 0.66 | 0.80 | 0.73 | 0.91 | 0.71 |
| Haryana | 85 | 60 | 91 | 51 | 95 | 94 | 91 | 0.81 | 0.55 | 0.57 | 0.52 | 0.82 | 0.78 | 0.95 | 0.71 |
| Himachal Pradesh | 72 | 70 | 90 | 67 | 88 | 87 | 86 | 0.51 | 0.70 | 0.51 | 0.77 | 0.58 | 0.48 | 0.82 | 0.62 |
| Jammu and Kashmir | 87 | 81 | 92 | 30 | 92 | 95 | 84 | 0.84 | 0.86 | 0.69 | 0.19 | 0.73 | 0.80 | 0.77 | 0.70 |
| Jharkhand | 68 | 39 | 91 | 28 | 76 | 83 | 69 | 0.41 | 0.22 | 0.58 | 0.17 | 0.14 | 0.30 | 0.38 | 0.31 |
| Karnataka | 71 | 71 | 94 | 45 | 97 | 94 | 87 | 0.48 | 0.71 | 0.85 | 0.42 | 0.90 | 0.75 | 0.85 | 0.71 |
| Kerala | 94 | 79 | 95 | 80 | 100 | 100 | 93 | 1.00 | 0.83 | 1.00 | 0.96 | 1.00 | 1.00 | 1.00 | 0.97 |
| Madhya Pradesh | 75 | 58 | 95 | 51 | 91 | 89 | 84 | 0.58 | 0.51 | 0.98 | 0.52 | 0.67 | 0.57 | 0.75 | 0.66 |
| Maharashtra | 71 | 70 | 90 | 48 | 95 | 94 | 85 | 0.48 | 0.70 | 0.52 | 0.48 | 0.82 | 0.75 | 0.80 | 0.65 |
| Odisha | 77 | 78 | 95 | 61 | 92 | 92 | 88 | 0.61 | 0.82 | 1.00 | 0.67 | 0.73 | 0.67 | 0.87 | 0.77 |
| Punjab | 69 | 59 | 90 | 55 | 94 | 96 | 86 | 0.42 | 0.54 | 0.48 | 0.59 | 0.80 | 0.82 | 0.82 | 0.64 |
| Rajasthan | 76 | 55 | 93 | 34 | 95 | 96 | 85 | 0.60 | 0.48 | 0.83 | 0.26 | 0.82 | 0.82 | 0.79 | 0.66 |
| Tamil Nadu | 77 | 90 | 90 | 83 | 100 | 100 | 93 | 0.63 | 1.00 | 0.48 | 1.00 | 0.99 | 0.99 | 1.00 | 0.87 |
| Telangana | 89 | 70 | 90 | 58 | 97 | 94 | 88 | 0.88 | 0.70 | 0.47 | 0.62 | 0.90 | 0.74 | 0.85 | 0.74 |
| Uttar Pradesh | 63 | 42 | 92 | 22 | 83 | 85 | 72 | 0.28 | 0.28 | 0.71 | 0.08 | 0.41 | 0.39 | 0.45 | 0.37 |
| Uttarakhand | 69 | 62 | 94 | 47 | 83 | 84 | 78 | 0.43 | 0.57 | 0.85 | 0.45 | 0.40 | 0.35 | 0.61 | 0.52 |
| West Bengal | 73 | 76 | 95 | 63 | 92 | 94 | 68 | 0.52 | 0.79 | 0.94 | 0.69 | 0.71 | 0.76 | 0.35 | 0.68 |

Note: Normalised value: Y_i_ = (X_i_ -X_min_) / (X_max_- X_min_), where Y_i_ is the normalised indicator for state i, X_i_ is the corresponding pre-normalisation value of the selected indicator, and X_max_ and X_min_ are the maximum and minimum values of the same indicator

Index=average of all the normalised value of indicators

**S 5.2: Index Value of Maternal Health Care: Major States (2019–21)** [29]

| State | Actual Value | | | | | | | Normalised value | | | | | | | Index |
| --- | --- | --- | --- | --- | --- | --- | --- | --- | --- | --- | --- | --- | --- | --- | --- |
|  | First Trimester  ANC | ANC 4 | TT2 | IFA  100 Days | Institutional  Delivery | Skilled  Assistance | PPC-2  Days | First Trimester  ANC | ANC 4 | TT2 | IFA  100 Days | Institutional  Delivery | Skilled  Assistance | PPC-2  Days |  |
| Andhra Pradesh | 82.4 | 76.3 | 95.0 | 56.2 | 91.6 | 92.2 | 79.7 | 0.80 | 0.82 | 0.95 | 0.81 | 0.80 | 0.77 | 0.81 | 0.82 |
| Assam | 55.1 | 46.5 | 89.9 | 32.0 | 70.6 | 74.3 | 54.0 | 0.36 | 0.43 | 0.77 | 0.39 | 0.29 | 0.24 | 0.28 | 0.40 |
| Bihar | 34.6 | 14.4 | 89.6 | 9.7 | 63.8 | 70.0 | 42.3 | 0.03 | 0.01 | 0.76 | 0.01 | 0.12 | 0.11 | 0.04 | 0.16 |
| Chhattisgarh | 70.8 | 59.1 | 94.3 | 30.3 | 70.2 | 78.0 | 63.6 | 0.61 | 0.59 | 0.92 | 0.36 | 0.28 | 0.35 | 0.48 | 0.51 |
| Delhi | 63.3 | 68.6 | 89.9 | 49.9 | 84.4 | 86.9 | 62.6 | 0.49 | 0.72 | 0.77 | 0.70 | 0.62 | 0.61 | 0.46 | 0.63 |
| Gujarat | 73.9 | 70.6 | 86.8 | 36.8 | 88.7 | 87.3 | 63.4 | 0.66 | 0.74 | 0.67 | 0.48 | 0.73 | 0.63 | 0.48 | 0.63 |
| Haryana | 63.2 | 45.1 | 92.3 | 32.5 | 80.5 | 84.7 | 67.3 | 0.49 | 0.41 | 0.86 | 0.40 | 0.53 | 0.55 | 0.56 | 0.54 |
| Himachal Pradesh | 70.5 | 69.1 | 86.3 | 49.4 | 76.4 | 78.9 | 70.2 | 0.60 | 0.72 | 0.65 | 0.69 | 0.43 | 0.38 | 0.62 | 0.59 |
| Jammu & Kashmir | 76.8 | 81.4 | 87.5 | 30.2 | 85.7 | 87.6 | 74.9 | 0.71 | 0.88 | 0.69 | 0.36 | 0.65 | 0.63 | 0.72 | 0.66 |
| Jharkhand | 52.0 | 30.3 | 91.8 | 15.3 | 61.9 | 69.6 | 44.4 | 0.31 | 0.22 | 0.84 | 0.11 | 0.08 | 0.10 | 0.09 | 0.25 |
| Karnataka | 66.0 | 70.3 | 88.3 | 45.3 | 94.3 | 93.9 | 65.6 | 0.53 | 0.74 | 0.72 | 0.62 | 0.86 | 0.82 | 0.52 | 0.69 |
| Kerala | 95.1 | 90.2 | 96.5 | 67.1 | 99.9 | 100.0 | 88.7 | 1.00 | 1.00 | 1.00 | 1.00 | 1.00 | 1.00 | 1.00 | 1.00 |
| Madhya Pradesh | 53.1 | 35.7 | 89.8 | 23.6 | 80.8 | 78.1 | 55.0 | 0.33 | 0.29 | 0.77 | 0.25 | 0.54 | 0.35 | 0.31 | 0.40 |
| Maharashtra | 67.6 | 72.2 | 90.4 | 40.6 | 90.3 | 91.1 | 78.5 | 0.56 | 0.76 | 0.79 | 0.54 | 0.77 | 0.74 | 0.79 | 0.71 |
| Odisha | 64.1 | 62.0 | 94.5 | 36.5 | 85.4 | 86.6 | 73.3 | 0.50 | 0.63 | 0.93 | 0.47 | 0.65 | 0.60 | 0.68 | 0.64 |
| Punjab | 75.6 | 68.5 | 92.9 | 42.6 | 90.5 | 94.1 | 87.2 | 0.69 | 0.72 | 0.88 | 0.58 | 0.77 | 0.83 | 0.97 | 0.77 |
| Rajasthan | 63.0 | 38.5 | 89.7 | 17.3 | 84.0 | 86.6 | 63.7 | 0.48 | 0.32 | 0.77 | 0.14 | 0.61 | 0.60 | 0.48 | 0.49 |
| Tamil Nadu | 64.0 | 81.2 | 71.0 | 64.0 | 99.0 | 99.3 | 74.1 | 0.50 | 0.88 | 0.12 | 0.95 | 0.98 | 0.98 | 0.70 | 0.73 |
| Telangana | 83.1 | 75.0 | 89.1 | 52.8 | 91.5 | 91.4 | 81.8 | 0.81 | 0.80 | 0.75 | 0.75 | 0.80 | 0.75 | 0.86 | 0.79 |
| Uttar Pradesh | 45.9 | 26.4 | 86.6 | 12.9 | 67.8 | 70.4 | 54.0 | 0.21 | 0.17 | 0.66 | 0.06 | 0.22 | 0.13 | 0.28 | 0.25 |
| Uttarakhand | 53.5 | 30.9 | 91.4 | 24.9 | 68.6 | 71.2 | 54.8 | 0.33 | 0.23 | 0.82 | 0.27 | 0.24 | 0.15 | 0.30 | 0.33 |
| West Bengal | 54.9 | 76.5 | 95.4 | 28.1 | 75.2 | 81.7 | 61.1 | 0.35 | 0.82 | 0.96 | 0.33 | 0.40 | 0.46 | 0.43 | 0.54 |

Note: Normalised value: Y_i_ = (X_i_ -X_min_) / (X_max_- X_min_), where Y_i_ is the normalised indicator for state i, X_i_ is the corresponding pre-normalisation value of the selected indicator, and X_max_ and X_min_ are the maximum and minimum values of the same indicator

Index=average of all the normalised value of indicators

**S6: Maternal Mortality Ratio (per 100,000 live births)** [30]

|  | 2004-06 | 2007-09 | 2010-12 | 2011-13 | 2014-16 | 2015-17 | 2016-18 |
| --- | --- | --- | --- | --- | --- | --- | --- |
| Maternal Mortality  Rate | 254 | 212 | 178 | 167 | 130 | 122 | 113 |

**S7: Maternal Mortality Ratio (per 100,000 live births)** [30]

|  | 2004-06 | 2007-09 | 2010-12 | 2011-13 | 2014-16 | 2015-17 | 2016-18 |
| --- | --- | --- | --- | --- | --- | --- | --- |
| India | 254 | 212 | 178 | 167 | 130 | 122 | 113 |
| EAG and Assam | 375 | 308 | 257 | 246 | 188 | 175 | 161 |
| South | 149 | 127 | 105 | 93 | 77 | 72 | 67 |
| Others | 174 | 149 | 127 | 115 | 93 | 90 | 83 |
